# Determining specific profiles of patients at risk of relapsing

**DOI:** 10.1192/j.eurpsy.2024.115

**Published:** 2024-08-27

**Authors:** H. Andreu Gracia

**Affiliations:** Institute of Neurosciences, Hospital Clínic of Barcelona, Barcelona, Spain

## Abstract

**Abstract:**

Based on the available literature and the studies presented by the previous speakers, Dr. Andreu will provide a summary of predictive and protective factors associated with mood relapse or recurrence in bipolar disorder, with a special focus on the distinction between modifiable and non-modifiable factors and on the identification of specific phenotypes at higher risk of relapse. The speaker will also mention the role of psychotherapeutic and pharmacological treatments, and will summarize the available evidence regarding lithium response.

**Disclosure of Interest:**

None Declared

